# Macrophage polarization in osteoarthritis progression: a promising therapeutic target

**DOI:** 10.3389/fcell.2023.1269724

**Published:** 2023-10-26

**Authors:** Yanlei Zhang, Quanbo Ji

**Affiliations:** ^1^ Medical School of Chinese PLA, Beijing, China; ^2^ Department of Orthopedics, The General Hospital of Chinese PLA, Beijing, China

**Keywords:** osteoarthritis, synovitis, macrophage polarization, cell therapy, tissue repairing

## Abstract

Osteoarthritis (OA) is one of the leading causes of pain and disability in the elderly. Synovitis, cartilage destruction and osteophyte formation histologically manifest OA. Unfortunately, there is currently no effective therapy to delay its progression and the underlying mechanisms of OA require further exploration. Macrophage is a main cellular component of joint synovium. It is highly plastic and can be stimulated to polarize to different phenotypes, namely, the pro-inflammatory phenotype (M1) and the anti-inflammatory/tissue-repairing phenotype (M2). Ample evidence has demonstrated the vital roles of macrophages in the progression of OA. Imbalanced M1/M2 ratio is significantly related to OA severity indicating macrophage polarization might be a promising therapeutic target for OA. In this review, we summarized the involvements of polarized macrophages in synovitis, cartilage degradation, osteophyte formation and OA-related chronic pain. Promising therapies targeting macrophage polarization including the intra-articular cell/derivates-based therapy and the alternative non-invasive intervention such as photobiomodulation therapy were reviewed as well.

## 1 Introduction

Osteoarthritis (OA) is a prevalent and heterogeneous degenerative disease, which is the leading cause of pain among elderly (Miller et al., 2014). It is characterized by synovitis, cartilage degeneration and osteophyte formation, and usually results in pain, joint swelling, joint space narrowing, physical impairment and even disability (Martel-Pelletier et al., 2016). Over 300 million people worldwide are affected by OA (Atukorala et al., 2016; Roos and Arden, 2016; Hunter and Bierma-Zeinstra, 2019). Despite the high prevalence, unfortunately, it is still a big challenge to treat OA patients in clinic because there’s no disease-modified drug to delay OA development or cure it. The most commonly used treatments for OA at present include non-steroid anti-inflammatory drugs (NSAIDs) and corticosteroids, which are accompanied with an extensive economic burden, unavoidable side effects and unsatisfactory outcomes (da Costa et al., 2021; Katz et al., 2021; Khumaidi et al., 2022). The potential mediators governing the initiation and progression of OA are not totally clear. New insights into the underlying mechanisms of OA and targeted therapeutics are urgently required.

**FIGURE 1:**
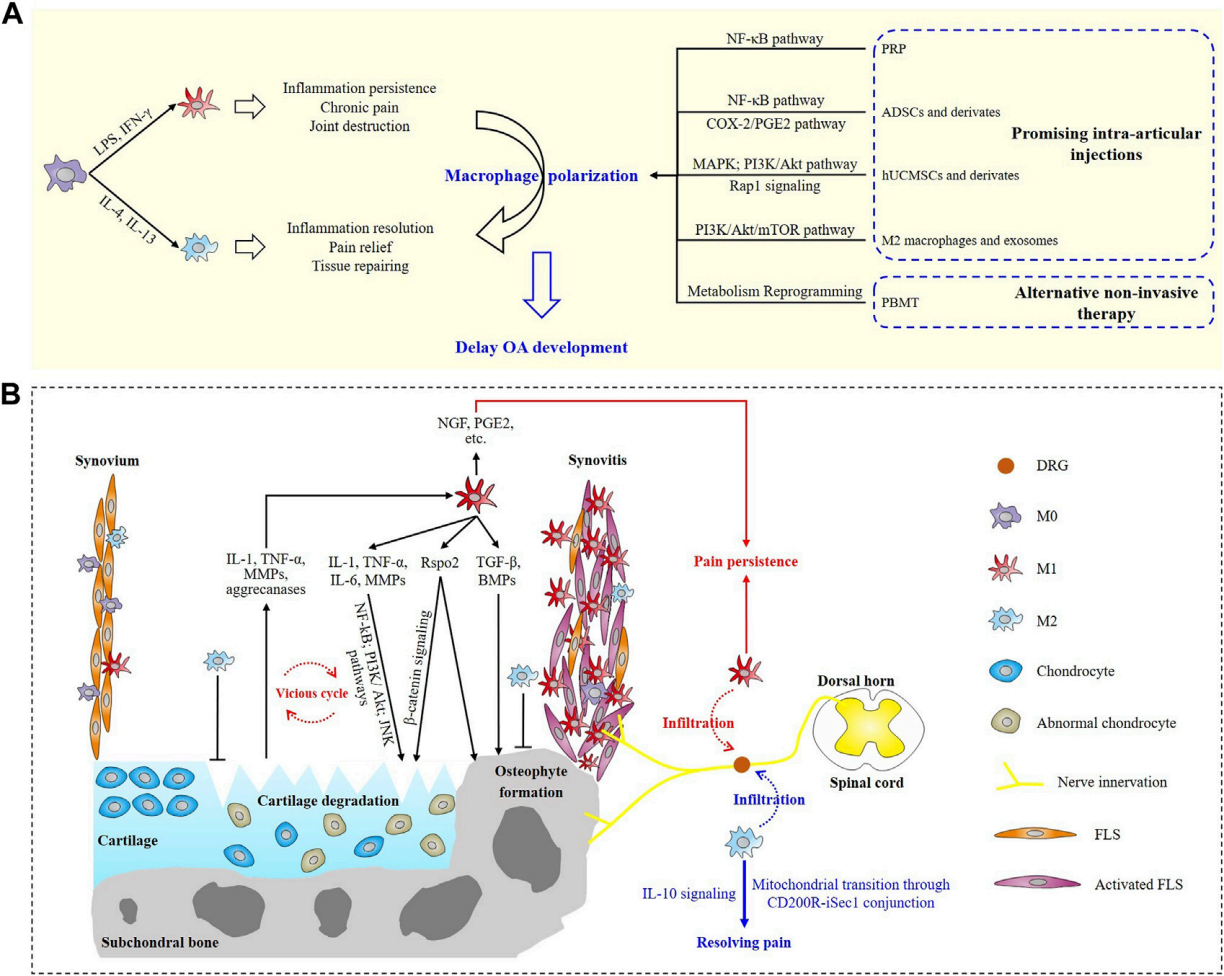
Schematic overview of macrophage polarization involved in osteoarthritis progression and potential treatments. **(A)** Roles of M1 and M2 macrophages in OA and promising macrophage polarization-targeted therapeutics. **(B)** Involvements of polarized macrophages in synovitis, cartilage degeneration, osteophyte formation and OA pain.

Macrophages scatter in many joint tissue components including synovium, ligament, bone marrow, subchondral bone, adipose tissue and so on. In physiological conditions, macrophages engulf pathogens and debris of aged tissues to maintain the microenvironment homeostasis of joint, while in OA joint, abnormally activated macrophages might worsen joint destruction (Zhang et al., 2018). Intriguingly, due to the highly plasticity of macrophages, they are demonstrated to be induced polarization to different phenotypes by different stimuli. For instance, macrophages can be polarized from an unstimulated M0 status to the pro-inflammatory M1 phenotype by lipopolysaccharide (LPS) and interferon-γ (IFN-γ), while interleukin (IL)-4/IL-13 is efficient to enhance the anti-inflammatory/tissue-repairing M2 phenotype macrophage polarization (Figure 1A) (Wynn and Vannella, 2016; Thomson and Hilkens, 2021). That is, to say, macrophages play a dual role in driving inflammation as well as promoting tissue regeneration. So far, ample evidence has demonstrated the pivotal roles of macrophage polarization in a variety of diseases including cancer (Li et al., 2023a), atherosclerosis (Tabas and Bornfeldt, 2016; Jinnouchi et al., 2020), osteoporosis (Wang et al., 2022), wound healing (Louiselle et al., 2021) and so on. In 2016, Kraus and colleagues (Kraus et al., 2016) provided the first direct *in vivo* evidence for macrophage involvements in human OA by applying a new ^99m^Tc-EC20 agent-based SPECT-CT imaging. Activated macrophages were found present in 76% of the knees. The quantity of knee-related activated macrophages was significantly associated with knee pain and radiographic knee OA severity including joint space narrowing and osteophyte formation, suggesting that macrophages-targeted interventions might work in attenuating OA progression. Subsequent experimental evidence from Zhang et al. (2018) showed that M1 but not M2-polarized macrophages accumulated in human and mouse OA synovium samples. Meanwhile, M1 macrophage polarization in synovium exacerbated collagenase-induced knee OA, shedding light on the potential of polarized macrophage as a promising therapeutic target for OA treatment.

In this review, we focused on summarizing the involvements of polarized macrophages in synovitis, cartilage degeneration, osteophyte formation and pain in OA. Promising therapies targeting macrophage polarization including intra-articular injections and alternative non-invasive interventions to OA were briefly introduced as well.

## 2 Pivotal roles of polarized macrophages in OA progression

### 2.1 Macrophage polarization and synovitis

OA has now been recognized as a whole-joint disease instead of a “wear and tear” disease induced by mechanical issues. Inflammation, synovitis in particular, significantly contributes to OA progression (Fernandez-Madrid et al., 1994; Benito et al., 2005). Cumulative evidence collectively proves the strong correlations between synovitis and OA symptoms, disease severity as well as outcomes (Ayral et al., 2005; Baker et al., 2010; Bacon et al., 2020), indicating synovitis may be a predictor or precursor of OA progression.

Macrophage and fibroblast-like synoviocyte (FLS) are the two main cellular components of synovium (Figure 1B). In OA joint, a large number of macrophage aggregation in the intimal layer of synovium manifests synovitis and it is suggested to drive the subsequent cartilage degeneration and osteophyte formation (Zhang et al., 2018). Kraus and colleagues preliminarily evaluated the involvements of the two macrophage phenotypes by performing immunostaining on synovial fluid samples of two patients, and the results suggested a potential block in the transition of macrophages from M1 to M2 phenotype (Kraus et al., 2016). This block might prolong the ongoing joint degeneration and prevents tissue repairing in OA, but it requires confirmation in a larger sample clinical trial. Liu et al. (2018) explored the status of M1 and M2 macrophages in knee OA patients for the first time. They collected the samples of both synovial fluids and peripheral whole blood from 80 knee OA patients and 80 healthy controls. M1 macrophages were shown to predominantly exist in OA knees and the ratio of M1/M2 macrophages was remarkably higher in OA knees and was significantly associated with Kellgren-Lawrence (KL) grading level, which indicated the imbalanced ratio of M1/M2 macrophages might be a sign of knee OA damage and severity (Liu et al., 2018). Actually, it’s further certified that M1 rather than M2 macrophages predominantly aggregated in the synovium of both OA patients and OA experimental animals, and M1 macrophages secreted R-spondin-2 (Rspo2) to exacerbate experimental OA progression (Zhang et al., 2018). Downregulating the polarization of M1 macrophages or abolishing the expression of Rspo2 may be a potential therapeutic target for OA.

To further evaluate the roles of polarized macrophages, macrophage depletion, which was considered a potential anti-OA intervention, was performed in experimental animals. By peritoneally injecting a small molecule (AP20187) in macrophage Fas-induced apoptosis (MaFIA)-transgenic mice, macrophages can be conditionally deleted (Burnett et al., 2004). Using this method, Wu et al. (2017) investigated the roles of macrophages in MaFIA knee OA mice in the setting of obesity. In this study, the injection of AP20187 was later demonstrated to successfully reduce the number of both M1 and M2 macrophages in the knees of OA mice compared to controls. However, systemic macrophage depletion did not attenuate the severity of OA in obese mice as expected; instead, it induced systemic inflammation, led to an extensive infiltration of CD3^+^ T cells and neutrophils, and remarkably increased the amount of pro-inflammatory factors; thereby exacerbated synovitis (Wu et al., 2017). This result was intriguing and it showed that the inflammatory state in macrophage-depleted OA mice transited from macrophage-associated to neutrophil-dependent. That is, to say, macrophage may be a double-edged sword in regulating OA synovitis. However, whether the failure of macrophage phenotype transition from M1 to M2 accounts for the post-depletion systemic inflammation is not concluded in this study. In another research, the effects of transient synovial depletion of macrophages on the inflammatory responses in post-trauma OA (PTOA) mice were evaluated (Bailey et al., 2020). Consistent with Wu’s research, intra-articular macrophage depletion 2 days prior to the joint injury induced intensive synovitis. In MaFIA mice, the depletion paradoxically altered macrophage polarization to a dominance of M1 phenotype within the joint synovium, while the expression of M2 macrophages in the synovial stroma was reduced (Bailey et al., 2020). The shift in M1/M2 macrophage ratio indicated macrophages might be critical immunomodulators in the acute inflammatory response to joint injury. These studies suggested that failure to correct polarity from the M1 to M2 subtype rather than the quantity of M1 or M2 macrophages may indeed mediate the progression and development of OA. But the precise mechanisms of macrophage phenotype transition in synovium warranted further investigation.

### 2.2 Macrophage polarization in cartilage degeneration

Irreversible cartilage loss is the main manifestation of OA. Activated macrophages mediate cartilage degradation and erosion mainly through autocrine and paracrine interactions. Both the inflammatory and destructive effects in OA joint are established to be largely dependent on synovial macrophages and these responses are driven by two main factors secreted by activated macrophages, namely, interleukin-1 (IL-1) and tumor necrosis factor-α (TNF-α) (Bondeson et al., 2006). Other potential mediators including matrix metalloproteinases (MMPs), cytokines, and growth factors were secreted into synovial fluid (SF) by activated synovial macrophages, FLS and damaged chondrocytes. The debris of destructed cartilage and mediators (MMP-9, MMP-13, aggrecanases, *etc.*) released by injured chondrocytes in turn facilitates M1 polarization by providing a proinflammatory microenvironment (Samavedi et al., 2017; Kulkarni et al., 2022). This interaction between synovial macrophages and chondrocytes provokes a vicious cycle that intensifies cartilage destruction and synovitis (Figure 1B) (Yin et al., 2022). CD14^+^ (a marker of M1 macrophage) synovial macrophages were shown to drive this effect. After specifically depleting CD14^+^ synovial macrophages *in vitro*, the synovial macrophages no longer produced significant amounts of macrophage-related cytokines (IL-1 and TNF-α), meanwhile, FLS-related mediators (IL-6, IL-8, MMP-1, and MMP-3) were also strongly downregulated (Bondeson et al., 2006). Activated macrophages can also lead to abnormal secretion of chondrocytes. The expression of MMP-1, MMP-3, MMP-9, MMP-13, IL-1β, TNF-α, IL-6, IL-8, and IFN-γ were observed to be significantly higher when chondrocytes were cocultured with activated macrophages, suggesting proinflammatory macrophages promoted the abnormal matrix degradation and chondrocyte-associated cytokine secretion (Samavedi et al., 2017). Mahon et al. (2020) treated human monocyte derived macrophages with basic calcium phosphate (BCP) crystals, known as OA-associated damage-associated molecular patterns (DAMPs), and assessed the expression of M1 and M2-associated markers. Treatment with BCP crystals promoted M1 macrophage polarization according to their results. Thereby, dampening the inflammatory responses provoked by M1 macrophages will definitely contribute to the amelioration of the vicious synovitis-cartilage degeneration cycle during OA development. Zhang et al. (2018) further explored the underlying mechanism for the macrophage-driving cartilage degeneration. In their study, tuberous sclerosis complex 1 (TSC1) deletion in mice was demonstrated to constitutively activate mechanistic target of rapamycin complex 1 (mTORC1), increase synovial M1 macrophage polarization and in this way exacerbate knee OA in experimental mice. And this effect was partially mediated through secretion of Rspo2 and activation of β-catenin signaling in chondrocytes (Figure 1B). Besides, inhibition of mTORC1 enhanced M2 polarization and in this way alleviated OA development in mice (Zhang et al., 2018). To date, several signaling pathways, including nuclear factor κB (NF-κB), PI3K/Akt, JNK and so on, are established participating in the cartilage degeneration of OA (Zhang et al., 2020; Zhao et al., 2023), however, associations between macrophage polarization and these pathways during OA pathogenesis are still under exploration.

### 2.3 Macrophage polarization in osteophyte formation

Osteophyte formation is a hallmark for advanced or end-stage OA. Systemic depletion of macrophages was capable of significantly reducing osteophyte formation in knee OA mice (Wu et al., 2017). Dominant M1 macrophages in synovium are shown to be associated with decreased bone mineral density (BMD) in macrophage-depleted MaFIA PTOA mice, suggesting M1 macrophages might be detrimental for bone homeostasis (Bailey et al., 2020). The secretion of growth factors such as transforming growth factor-β (TGF-β) and bone morphogenetic protein (BMP) is established to be responsible for regulating osteophyte formation in OA, because they are efficient in promoting osteogenesis, chondrogenesis and osteogenic differentiation of mesenchymal stem cells (MSCs) (Blom et al., 2004; Zhang et al., 2020). By establishing a collagenase-induced OA (CIOA) model, Blom et al. (2004) investigated the involvements of synovial macrophages in osteophyte formation and demonstrated macrophages provoke osteophyte formation through regulating the expressions of TGF-β and BMPs (Figure 1B). In their study, prior to OA induction, macrophages were selectively removed using clodronate liposomes. Depletion of synovial macrophages resulted in spectacular reduction of osteophyte formation, fibrosis and synovial activation were also significantly ameliorated. In addition, production of TGF-β, BMP-2 and BMP-4 in the lining was largely prevented but production of them in deeper layers of the synovium and the periosteum did not differ from controls. Other cytokines like IL-1β and TNF-α are able to activate osteoclast formation and promote bone resorption, which exacerbates osteophyte formation in OA (Zhang et al., 2020). Intra-articular injections of recombinant human IL-1 receptor antagonist (IL-1Ra) were demonstrated protective in inhibiting the development of osteophytes and cartilage lesions in an experimental canine OA model, showing the positive effects of functional suppression of pro-inflammatory macrophages in OA osteophyte formation (Caron et al., 1996). In addition, M1 macrophages were also shown to secret Rspo2 to exacerbate osteophyte formation and worsen OA progression (Zhang et al., 2018). All these findings suggest the crucial roles of polarized macrophages in osteophyte formation.

### 2.4 Macrophage polarization and OA pain

Pain is the first and most common symptom in OA patients, and is the main reason that patients seek for medical assistance. 25% of adults over 55 years old experience knee pain at least once a year, which may be a marker of potential knee OA (Shamsi et al., 2021). The current interventional strategies for OA dominantly aim at relieving pain and improving functions. However, the existing pain management methods are inadequate and unsatisfactory. NSAIDs are the most frequently used and are recommended in most of the clinical guidelines for OA, but it’s accompanied with potential risks of side effects, for instance gastrointestinal toxicity and bleeding; while opiates bring risks of addiction (Conaghan et al., 2019; Hunter and Bierma-Zeinstra, 2019; Stausholm et al., 2019). Total joint replacement surgery is thought to be the most effective method for advanced and end-stage OA patients, however, about 7%–23% patients after hip replacement and 10%–34% after knee replacement reported an unfavorable long-term pain (Beswick et al., 2012). Therefore, it’s essential to figure out the mechanisms of OA pain in order to provide new insights for developing effective targeted pain management interventions. Macrophages might be an etiologic factor for OA pain because joint pain is shown to be significantly positively associated with the presence of activated macrophages in OA joint (Kraus et al., 2016).

It has been established that tissue injury, inflammatory response and central sensitization collectively contribute to OA-related pain (Raoof et al., 2018; Conaghan et al., 2019). M1 macrophages are illuminated to mediate OA pain through regulating local secretion of pain-related inflammatory factors including IL-1β, TNF-α, prostaglandin E2 (PGE2) *etc.*, which not only promote synovitis and joint destruction, but also directly activate innervating nociceptors (Miller et al., 2014; Conaghan et al., 2019). Meanwhile, macrophage-derived IL-1β and TNF-α can also regulate the expression of nerve growth factor (NGF) in synovial macrophages, indicating the development and persistence of OA pain (Figure 1B) (Takano et al., 2017).

Central sensitization gives an explanation for why approximately 7%–34% of patients reported chronic post-surgical pain after the receiving total joint arthroplasty surgery and why OA pain is often found to be not always associated with radiographic manifestations. Apart from the secretion of pro-inflammatory factors, polarized macrophages also involve in central sensitization of OA pain. Raoof et al. (2021) identified that dorsal root ganglion (DRG) containing the somata of sensory neurons innervating the damaged knee were infiltrated with M1 macrophages. These M1 macrophages in DRG actively maintained OA joint pain remotely and independent of joint damage. Inhibiting M1 macrophages in DRG by intrathecal injection of IL4-10 fusion protein or M2 macrophages resolved persistent OA pain. Their work reveals a novel mechanism that help to maintain OA pain distant from the affected knee and suggests that DRG macrophages will be a new therapeutic target to treat OA chronic pain. In their later study, M2 macrophages were found to infiltrate in the DRG containing the somata of sensory neurons during the resolution of inflammatory pain. And M2 phenotype macrophages could actively control OA inflammatory pain resolution remotely from the site of inflammation by transferring mitochondria to sensory neurons, which was mediated by conjunction of CD200 receptor (CD200R) on macrophages and iSec1 (a non-canonical CD200R ligand) on sensory neurons. This research revealed a novel and direct mechanism for M2 macrophages in actively resolving inflammatory pain (van der Vlist et al., 2022). In addition to the direct participation of macrophages in OA pain resolution, monocytes/macrophages are uncovered to resolve the inflammatory hyperalgesia via IL-10 signaling-dependent mechanism in DRG (Figure 1B), suggesting the cardinal roles of M2 macrophages in pain resolution (Willemen et al., 2014).

## 3 Promising macrophage polarization-targeted anti-OA therapy

Macrophage polarization sheds lights on developing new anti-OA interventions. To date, compelling experiments have been performed to test the effects of different anti-OA therapeutics, including medicine, cell therapy, nanomaterials, surgery, *etc.*, on driving macrophage polarization (Ma et al., 2021; Robb et al., 2022; Li et al., 2023b; Liu et al., 2023; Zheng et al., 2023; Zhou et al., 2023). All of these preclinical researches are shown to be effective in inhibiting pro-inflammatory cytokines release, suppressing M1 polarization and enhancing M2 polarization. Herein, we simply focused on promising intra-articular cells/derivates-based therapies and alternative non-invasive interventions that target macrophage polarization for treating OA.

### 3.1 Future intra-articular injection of cells and derivates targeting polarized macrophages

Intra-articular injection of medicine, including glucocorticoid, has long been applied for treating OA patients. It is effective by providing short-term analgesia and anti-inflammatory effects (Li et al., 2022a). However, long-term repeated injections will cause loss of cartilage or injury of other joint components. Therefore, to investigate bio-modulatory medium that are able to meanwhile promote cartilage proliferation and modulate joint homeostasis will definitely be necessary and useful for developing new intra-articular therapeutics.

Cells (usually stem cells) derived from adipose, umbilical cord and bone marrow, *etc.*, or their derivates including extracellular vesicles (EVs) and exosomes are now under extensive investigation for treating OA. One example that has been increasingly applied in clinic is the platelet-rich plasma (PRP). PRP is derived from autologous blood and is suggested to maintain the homeostasis of joints by providing an analgesic effect, facilitating inflammation resolving and promoting anabolic metabolism (Uchiyama et al., 2021). Preclinical experiments have demonstrated that PRP suppressed M1 macrophage polarization and promoted M2 macrophage polarization in OA (Uchiyama et al., 2021). PRP exerts anti-inflammatory properties through its effects on the canonical NF-κB signaling pathway in multiple cell types including synoviocytes, macrophages and chondrocytes (Andia and Maffulli, 2013). A series of randomized clinical trials (RCTs) and meta-analysis have been performed to investigate the efficacy and safety of intra-articular PRP injections towards OA, however, the current findings do not really support the use of PRP injections for knee or ankle OA patients (Bennell et al., 2021; Paget et al., 2021).

Adipose-derived mesenchymal stem cells (ADMSCs) are indicated as one of the most promising cell sources, and they may exert their powerful immunomodulatory and anti-inflammatory activities through the cyclooxygenase-2 (COX-2)/prostaglandin E2 (PGE2) pathway (Manferdini et al., 2013). Besides, NF-κB1 and NF-κBIA (the NF-κB signaling inhibitor) were significantly decreased by ADMSC treatment, suggesting ADMSCs potentially switching off macrophages’ activity strictly dependent on NF-κB signaling (Paolella et al., 2019). The therapeutic potential of EVs derived from human adipose-derived stem cells (hADSCs) in alleviating OA was evaluated (Woo et al., 2020). Intra-articular injection of hADSC-EVs significantly attenuated OA progression and protected cartilage from degeneration in knee OA mouse models by inhibiting M1 macrophages infiltration in the synovium. KEGG pathway enrichment analysis indicated “ECM-receptor interaction”, and “glycosphingolipid biosynthesis–lacto and neolacto series” as dominant pathways controlled by hADSC-EVs (Woo et al., 2020). Both human umbilical cord mesenchymal stem cells (hUCMSCs) and EVs derived from hUCMSCs are efficient to promote M2 macrophage polarization, inhibit M1 macrophage expression (Tang et al., 2021). Notably, compared with hUCSMCs, small EVs derived from hUCMSCs exert stronger effects in maintaining cartilage homeostasis and alleviating cartilage damage by upregulating proteins involved in immune effector process, extracellular matrix organization, PI3K-Akt and Rap1 signaling pathway (Tang et al., 2021). In another study, hUCMSCs-EVs can alleviate cartilage degradation during the OA progression, mechanically may through modulating the PI3K-Akt signaling pathway mediated by miRNAs to promote polarization of M2 macrophage and exhibit the potent immunomodulatory potential (Li et al., 2022b). Besides, exosomes derived from hUCMSC resist IL-1β or M1 macrophages-induced chondrocyte inflammation dominantly through the MAPK and PI3K-Akt signaling pathways, which was demonstrated by performing a KEGG pathway enrichment analysis (Wang et al., 2023). All of these results suggest exosomes or EVs derived from ADMSC or hUCMSC might serve as a new reagent for treating OA.

Recently, Ma et al. (2021) proposed and fabricated an intriguing artificial M2 macrophage (AM2M) with yolk-shell structure to enhance the therapeutic efficacy of M2 macrophages in treating OA. AM2M was composed of macrophage membrane as “shell” and inflammation-responsive nanogel as “yolk”. AM2M exhibited the targeting and long-term residence in the inflamed area and blocked the immune stimulation of macrophages, indicating AM2M might be a promising medium applied for future intra-articular therapies for OA (Ma et al., 2021). But the potent mechanical signaling pathways accounting for the effects of AM2M in OA are uncovered in this research. Apart from M2 macrophages themselves, exosomes derived from M2 macrophages also exert positive effects towards protecting articular cartilage and attenuating inflammatory responses in knee OA rats mainly through the PI3K/AKT/mTOR signal pathway (Da-Wa et al., 2021).

Preclinical experiments indicate the potential of intra-articular injections of cells and derivates in driving synovial macrophage polarization for OA joints. However, few clinical evidence has elucidated the presence of macrophage phenotype transition in OA patients. Cell and derivates-based therapies are promising, but unnecessary injuries might happen when injecting the cells or derivates into joints. Besides, larger-sample and long-term studies are mandatory to confirm whether these therapies can improve symptoms and induce structural benefits in OA patients.

### 3.2 Alternative non-invasive therapy targeting macrophage polarization

Photobiomodulation therapy (PBMT), also known as low-level light therapy (LLLT), is an intriguing intervention method using low-intensity laser or light emitting diodes (LEDs) with wavelengths in the range of visible (400–700 nm) or near-infrared (NIR, 700–1,000 nm) (Amaroli et al., 2019). PBM displays great advantages in treating OA because it is totally non-invasive with rarely reported side effects. *In vivo* studies have shown the efficacy of PBM in relieving pain, decreasing synovial inflammatory factors (IL-1β, TNF-α, IL-6 *etc.*) expression and attenuating joint swelling in OA animals (Alves et al., 2013; da Rosa et al., 2012; Trevisan et al., 2020; Wang et al., 2014; Yamada et al., 2020). In Zhang et al. (2022) latest study, they provided the first *in vivo* evidence for the efficacy of PBMT in modulating synovial macrophage polarization in collagenase-induced knee OA mice. A 630 nm Light Emitting Diode (LED) device was applied to illuminate the right knees of OA mice with different power densities. After 4-week consecutive treatments (1 hour irradiation per time per day), the pain and gait of OA mice were significantly attenuated in the group irradiated with a power density of 10 mW/cm^2^. Histological analysis and immunostaining results suggested a great amelioration of synovitis, cartilage degeneration and reduction of osteophyte formation in experimental mice, which might be partially mediated by the synovial macrophage polarity transition from M1 to M2 phenotype. Unfortunately, they didn’t further explore the potential mechanistic pathways of the phenotype transition caused by PBMT. Metabolically, inflammatory M1 macrophages display enhanced glycolytic metabolism, conversely, anti-inflammatory M2 macrophages show high mitochondrial oxidative phosphorylation (OXPHOS) (Van den Bossche et al., 2015). PBMT is believed to be sufficient in enhancing mitochondrial OXPHOS by increasing mitochondrial activity and ATP production (Amaroli et al., 2019; Zhang and Qu, 2023). Given this, metabolism reprogramming induced by light irradiation might be a potential cellular mechanism for PBMT in enhancing macrophage polarization and treating OA (Figure 1A). But it still requires further experimental verification in OA animals and patients.

## 4 Conclusion and future perspectives

It’s now widely acknowledged that OA is a multifactorial disease. Synovitis and macrophages play primordial roles in the onset and progression of OA. Macrophages function as a bridge between synovium, cartilage, adipose tissue, and subchondral bone. The cellular interactions among polarized macrophage, chondrocyte, FLS, *etc.*, contribute to both homeostasis maintenance and pathological destruction of joint. Targeting macrophage polarization provides new insights for breaking the vicious and self-perpetuating cycle of OA. Future studies should focus on the following topics. First, detailed interactions between macrophages and other cellular components in OA joint should be uncovered. Second, the concise subsets of macrophages that participate in the whole process of OA progression need more clarification. Finally, understanding the underlying mechanisms of macrophage phenotypes transition and developing alternative targeted therapeutic methods. Larger sample clinical trials are required to evaluate the existence of macrophage polarization in human body.
